# Behandlung von Hepatitis-C-Infektionen im Zeitalter direkt wirkender antiviraler Medikamente (DAAs)

**DOI:** 10.1007/s00103-021-03481-z

**Published:** 2022-01-10

**Authors:** Kai-Henrik Peiffer, Stefan Zeuzem

**Affiliations:** Medizinische Klinik 1, Universitätsklinikum Frankfurt, Theodor-Stern-Kai 7, 60590 Frankfurt am Main, Deutschland

**Keywords:** HCV, DAA, Interferonfrei, SVR, Antivirale Therapie, HCV, DAA, Interferon-free, SVR, Antiviral treatment

## Abstract

Die chronische Hepatitis-C-Infektion kann unbehandelt zu schwerwiegenden und potenziell lebensbedrohlichen leberassoziierten Komplikationen führen. Grundsätzlich stellt damit jede chronische Infektion mit dem Hepatitis-C-Virus (HCV) eine Indikation zur antiviralen Therapie dar. Besonders dringlich ist sie jedoch bei Patient*innen mit fortgeschrittener Lebererkrankung. In diesem Beitrag werden Indikation, Therapieziele und Grundprinzipien der direkt antiviralen Therapie beschrieben. Verschiedene Therapieregime und Möglichkeiten der Überwachung von Therapie und Therapieerfolg werden vorgestellt.

Heutzutage wird die chronische HCV-Infektion interferonfrei mit direkt antiviral wirksamen Medikamenten („direct acting antivirals“ – DAA) behandelt, wobei die Wahl der Medikamente von HCV-Genotyp, Vortherapie und Fibrosestatus abhängt. Patient*innen mit kompensierter Leberzirrhose und solche ohne Leberzirrhose weisen unter Behandlung vergleichbar hohe Viruseradikationsraten auf. Auch bei dekompensierter Leberzirrhose oder dialysepflichtiger Niereninsuffizienz und selbst bei Kindern ab einem Alter von 3 Jahren ist heutzutage eine sichere und hocheffiziente antivirale Behandlung möglich. Medikamenteninteraktionen sind zu beachten, können aber einfach und schnell im Internet überprüft werden. Auch wenn sich die Prognose nach HCV-Eradikation deutlich verbessert, sollten Patient*innen mit fortgeschrittener Leberfibrose bzw. einer Leberzirrhose lebenslang weiterbeobachtet werden, um die Entstehung eines hepatozellulären Karzinoms rechtzeitig zu erkennen (HCC-Surveillance).

## Abstract (en)

Untreated chronic hepatitis C infection can lead to severe and potentially fatal liver-associated complications. Therefore, every hepatitis C virus (HCV) infection represents an indication for antiviral treatment. In particular, patients with progressive liver disease should be treated urgently. Here, we review indication for treatment as well as goals and basic principles of antiviral therapy. In addition, different treatment regimens and monitoring of the treatment course and outcome are discussed.

Today, the treatment of chronic HCV infection is based on interferon-free regimens combining different direct-acting antivirals (DAAs), where the choice of DAA-regimen depends on the viral genotype, previous treatments, and the state of liver fibrosis. With these regimens, equally high virus eradication rates are achievable in patients with compensated liver cirrhosis and in patients without advanced liver disease. In addition, patients with decompensated liver cirrhosis or patients with end-stage renal failure requiring renal replacement therapy, as well as children from an age of 3 years, can be treated safely and highly efficiently with DAA-containing regimens. Physicians should be aware of possible drug interactions of the DAAs with concomitant administered drugs. However, possible interactions can be checked easily online. Although, there is an improvement of prognosis after HCV eradication, patients with advanced liver fibrosis or liver cirrhosis must be included in a lifelong HCC surveillance program.

## Hintergrund

Eine Infektion mit dem hepatotropen Hepatitis-C-Virus kann unbehandelt zu schwerwiegenden und potenziell lebensbedrohlichen Komplikationen wie der Entwicklung einer Leberzirrhose und/oder eines hepatozellulären Karzinoms (HCC) führen. In der Zeit vor der Zulassung von direkt antiviral wirksamen Medikamenten (engl.: „direct acting antivirals“, DAAs) wurde durch die nebenwirkungsreiche, indirekt über das Immunsystem vermittelte Interferontherapie nur ein Teil der Infizierten geheilt. Durch die Einführung von DAAs wurde die antivirale Therapie revolutioniert. Heutzutage gibt es hochwirksame, interferonfreie Therapieregime mit Heilungsraten von > 95 % für alle Patient*innengruppen.

### Epidemiologie weltweit und in Deutschland

Laut Berechnungen der Weltgesundheitsorganisation (WHO) sind weltweit ca. 58 Mio. Menschen mit dem Hepatitis-C-Virus (HCV) chronisch infiziert, was beispielsweise 2019 zu 290.000 Todesfällen aufgrund HCV-assoziierter Komplikationen wie der Leberzirrhose und/oder eines HCC geführt hat [1]. Aufgrund der hohen Replikationsrate und der kurzen Lebensdauer von HCV konnte bisher trotz zahlreicher Bestrebungen noch keine ausreichend wirksame Vakzine entwickelt werden. Zudem ist trotz des Vorhandenseins hochwirksamer Therapieregime der weltweite Zugang zu Diagnostik und Therapie weiterhin stark begrenzt. In Deutschland bestehen keine Limitationen hinsichtlich Diagnostik und Therapie. Beispielweise haben in Deutschland Versicherte ab 35 Jahren seit 2020 nach dem Urteil des gemeinsamen Bundesausschuss (G-BA) das Anrecht auf ein einmaliges HCV-Screening mittels Antikörpertestung.

Laut Robert Koch-Institut ist die Anzahl gemeldeter HCV-Infektionen im Vergleich zu den Vorjahren 2018 und 2019 im Jahr 2020 deutlich um 24 % auf eine Inzidenz von 5,5/100.000 Einwohner*innen zurückgegangen. Ob es sich hierbei um einen tatsächlichen Rückgang der Neuinfektionen oder einen artifiziellen Rückgang durch Untererfassung bzw. Rückgang der Diagnostik im Rahmen der COVID-19-Pandemie handelt, lässt sich aktuell nicht sicher belegen [2]. Die Gesamtprävalenz in Deutschland beträgt 0,3 % und ist damit niedrig im Vergleich zu anderen Regionen der Welt.

Die Transmission erfolgt vor allem durch gemeinsamen Spritzengebrauch, sogenanntes Needle Sharing beim Drogenabusus, homosexuelle Kontakte unter Männern sowie durch iatrogene Übertragung im Gesundheitswesen vor allem in Ländern mit mangelnder Einhaltung von Hygienerichtlinien.

### Krankheitsverlauf

Grundsätzlich wird eine akute von einer chronischen HCV-Infektion unterschieden, wobei man von einem chronischen Verlauf ausgeht, wenn die Infektion länger als 6 Monate besteht.

Während 50–70 % der akuten Infektionen chronifizieren, ist die Progression zur Fibrose abhängig vom Infektionsalter. In 30 Jahren entwickelt sich bei ca. 20–30 % der Patient*innen eine Leberzirrhose [3]. Die Fibroseprogression ist in hohem Maße abhängig von Kofaktoren, wie zum Beispiel zusätzlichem schädlichen Alkoholgebrauch und Koinfektion mit HIV oder anderen hepatotropen Viren. Bei bestehender Leberzirrhose entwickelt sich in 3–6 % der Patient*innen ein hepatozelluläres Karzinom (HCC).

## Indikation und Angriffspunkte der antiviralen Therapie

Grundsätzlich besteht bei jeder replikativen HCV-infektion die Indikation zu einer antiviralen Therapie, insbesondere wenn bereits eine fortgeschrittene Lebererkrankung oder extrahepatische Manifestationen bestehen [4]. Die Therapieindikation besteht prinzipiell auch für akute Infektionen, die aber in der klinischen Praxis häufig nicht sicher von einer chronischen Infektion differenziert werden können. Im Falle einer kürzlich erworbenen Infektion, die zum Beispiel bei HCV-RNA-Positivität und gleichzeitig noch bestehender Negativität für HCV-Antikörper angenommen wird, kann bei hoher Wahrscheinlichkeit für eine spontane Elimination und relativ niedrigem Risiko für eine Übertragung zunächst der Spontanverlauf abgewartet werden. Eine Postexpositionsprophylaxe nach Nadelstichverletzung im medizinischen Kontext wird aufgrund sehr niedriger HCV-Übertragungsraten bei Nadelstichverletzungen nicht empfohlen.

Das Hepatitis-C-Virus ist ein einzelsträngiges, umhülltes RNA-Virus aus der Familie der Flaviviridae. Das virale Genom codiert für mindestens 3 strukturbildende und 7 nichtstrukturbildende (NS) Proteine, von denen insbesondere die NS3-, NS5A- und NS5B-Proteine als Angriffspunkte für direkt antiviral wirksame Substanzen relevant sind. Aufgrund einer hohen genetischen Variabilität haben sich mindestens 6 verschiedene virale Genotypen und zahlreiche Subtypen herausgebildet, die sich zum Teil untereinander von dem durch sie bedingten Krankheitsverlauf und in der Ansprechrate auf eine antivirale Therapie unterscheiden [5].

## Therapieziele und Prognose

Das Therapieziel einer antiviralen Therapie ist eine dauerhafte Viruseradikation (SVR = „sustained virologic response“), die durch einen fehlenden Nachweis von HCV-RNA 12 Wochen nach Ende einer HCV-Therapie angezeigt wird. Da die Eradikation des Virus zu keiner protektiven Immunität führt, sind Neuinfektionen möglich. Eine Metaanalyse beschrieb beispielweise eine Reinfektionsinzidenz von 6,4 pro 100 Patientenjahren in Patient*innen mit aktivem intravenösen Drogengebrauch [6]. Prognostisch geht das Erreichen einer SVR mit einer deutlichen Senkung der Letalität, des Risikos für die Entwicklung eines HCC sowie der Notwendigkeit einer Lebertransplantation einher [3, 7].

Während diese positiven Effekte am deutlichsten bei Patient*innen im präzirrhotischen Stadium oder bei Vorhandensein einer kompensierten Leberzirrhose zu beobachten sind, besteht bei dekompensierter Leberzirrhose zum Therapiezeitpunkt trotz SVR weiterhin ein hohes Risiko für eine assoziierte Komplikation [8]. So zeigte beispielweise eine italienische Studie eine jährliche HCC-Rate von 7,8 % in Patient*innen mit einer fortgeschrittenen Child-Pugh-B-Leberzirrhose im Vergleich zu 2,1 % bei jenen mit child-pugh-A-klassifizierter Leberzirrhose [9].

## Direkt antivirale Medikamente (DAAs) und Grundprinzipien der Therapie

Die Therapie der Hepatitis C soll heutzutage mit einem interferonfreien Therapieregime durchgeführt werden [4, 10]. In diesen Regimen werden grundsätzlich direkt antivirale Medikamente kombiniert, die eine hohe antivirale Wirksamkeit bei gleichzeitig hoher Resistenzbarriere aufweisen und sich in ihren viralen Angriffspunkten unterscheiden.

Anhand ihrer molekularen Wirkweise werden DAAs in Polymeraseinhibitoren, Proteaseinhibitoren und NS5A-Inhibitoren eingeteilt. Polymeraseinhibitoren agieren durch eine kompetitive oder allosterische Hemmung der viralen NS5B-Polymerase und bewirken so einen Kettenabbruch der wachsenden viralen RNA. Die generischen Namen der zur Therapie zugelassenen HCV-Polymeraseinhibitoren enden auf „…-buvir“. Die Wirkung der Proteaseinhibitoren beruht auf einer verminderten Spaltung des HCV-Polyproteins durch Hemmung der viralen NS3/4A-Serinprotease. Die generischen Namen dieser Medikamentenklasse enden auf „…-previr“. NS5A-Inhibitoren wiederum hemmen das virale NS5A-Protein, welches funktionell an der HCV-Replikation und Modulation zellulärer Funktionen beteiligt ist. Die generischen Namen dieser Wirkstoffklasse enden auf „…-asvir“. (Für eine Übersicht der in Deutschland aktuell zugelassenen und erhältlichen DAAs siehe Tab. 1).

**Table 1:** 

Substanzklasse	Medikament	Dosierung (Erwachsene) pro Tag	HCV-Genotypen-Aktivität (*in vitro *und per Zulassung)
Nukleotidischer NS5B-Polymeraseinhibitor	Sofosbuvir^b,d,e^ (SOF)	400 mg	1a, 1b; 2a, 2b; 3a; 4a, 4b; 5a; 6a, 6e, 6p
			Zulassung: 1–6
NS3-Proteaseinhibitoren	Grazoprevir^a^ (GZR)	100 mg	1a, 1b; 4
			Zulassung: 1, 4
	Voxilaprevir^b^ (VOX)	100 mg	1a, 1b; 2a, 2b; 3a; 4a, 4d; 5a; 6a, 6e, 6n
			Zulassung: 1–6
	Glecaprevir^c^ (GPR)	3 × 100 mg	1a, 1b; 2a, 2b; 3a; 4a, 4d; 5a
			Zulassung: 1–6
NS5-Inhibitoren	Ledipasvir^d^ (LDV)	90 mg	1a, 1b; 2a, 2b; 3a; 4a, 4d, 5a, 6a, 6e
			Zulassung: 1, 4–6
	Velpatasvir^e^ (VEL)	100 mg	1a, 1b, 2a; 2b; 3a; 4a, 4d, 4r; 5a; 6a; 6e
			Zulassung: 1–6
	Elbasvir^a^ (EBR)	50 mg	1a, 1b; 4
			Zulassung: 1, 4
	Pibrentasvir^b^ (PIB)	3 × 40 mg	1a, 1b; 2a, 2b; 3a; 4a, 4d; 5a
			Zulassung: 1–6

*HCV* Hepatitis-C-Virus, *DDA* „direct acting antivirals“

^a^Koformulierung pro Tablette: 100 mg Grazoprevir, 50 mg Elbasvir

^b^Koformulierung pro Tablette: Sofosbuvir, 100 mg Velpatasvir, 100 mg Voxilaprevir

^c^Koformulierung pro Tablette: 100 mg Glecaprevir, 40 mg Pibrentasvir

^d^Koformulierung pro Tablette: 400 mg Sofosbuvir, 90 mg Ledipasvir

^e^Koformulierung pro Tablette: 400 mg Sofosbuvir, 100 mg Velpatasvir

Als Kombinationspartner für DAAs ist in einigen Therapieregimen zudem weiterhin der Wirkstoff Ribavirin vertreten, welcher kein direkt antiviral wirksames Medikament ist und bereits in der Interferonära als Kombinationspartner genutzt wurde. Die antivirale Wirkweise des Ribavirins ist in vielen Teilen unverstanden [3]. Ribavirin hat nachgewiesenermaßen jedoch auch antivirale Wirksamkeit gegen andere hepatotrope Viren, wie zum Beispiel das Hepatitis-E-Virus.

## Verschiedene Therapieregime

Nach Einführung der beiden Proteaseinhibitoren Telaprevir und Boceprevir im Jahr 2011 als erste zugelassene DAAs [11, 12] haben sich die therapeutischen Optionen in den letzten 10 Jahren grundlegend weiterentwickelt. Aktuell stehen für alle HCV-Genotypen und nahezu alle Patient*innenkollektive hocheffektive Therapieregime zur Verfügung, mit denen eine SVR in > 95 % der Fälle erreicht werden kann. Grundsätzlich müssen bei der Auswahl des Therapieregimes und der Therapiedauer der HCV-Genotyp, eine gegebenenfalls durchgeführte Vortherapie sowie das Fibrose‑/Leberzirrhosestadium berücksichtigt werden. Die Bestimmung des Fibrosestadiums kann heutzutage mittels nichtinvasiver biochemischer Algorithmen wie dem AST/Thrombozyten-Ratio-Index (APRI) oder dem Fibrosis‑4 Index (FIB-4) oder mittels elastografischer Verfahren erfolgen. Eine Leberbiopsie zur Fibrosebestimmung ist daher in der Regel nicht mehr erforderlich.

Für die Therapie der chronischen Hepatitis-C-Infektion sind in Deutschland verschiedene koformulierte, fixe DAA-Kombinationsregime zugelassen. Zu unterscheiden sind pangenotypisch (für alle Genotypen) wirksame Therapieregime und genotypspezifische Therapieregime [4]:

Pangenotypisch wirksam:

- Sofosbuvir/Velpatasvir
- Glecaprevir/Pibrentasvir

Genotypspezifisch (Genotyp 1 und 4) wirksam:

- Sofosbuvir/Ledipasvir
- Grazoprevir/Elbasvir

Für die Auswahl eines Regimes sind zum einen medizinische (Genotyp/Vortherapie/Fibrosestadium), zum anderen aber auch wirtschaftliche Kriterien relevant. Weiterhin müssen bei der Therapieauswahl Komorbiditäten, wie zum Beispiel eine gegebenenfalls vorhandene Niereninsuffizienz, und mögliche Medikamenteninteraktionen berücksichtigt werden. Bei sogenannten leicht zu behandelnden Patient*innen kann zudem eine hocheffektive antivirale Therapie auch ohne Bestimmung des viralen Genotyps mit einem der beiden pangenotypischen Therapieregime durchgeführt werden (für eine Übersicht zur leitliniengemäßen Einsetzbarkeit der pangenotypischen Regime siehe Tab. 2). Als leicht zu behandeln gelten hierbei Patient*innen 1. ohne Vortherapie mit einem DAA-Kombinationsregime, 2. ohne dekompensierte Leberzirrhose (Child-Pugh-B/C) und 3. ohne fortgeschrittene Niereninsuffizienz (glomeruläre Filtrationsrate < 30 ml/min; [4]).

**Table 2:** 

Therapieregime	Therapiedauer in Wochen	Patient*innen ohne Leberzirrhose		Patient*innen mit kompensierter Leberzirrhose		
		TN^a^/TE^b^	HCV Gt3 + TE^c^	TN^a^	TE^b^	HCV Gt3 + TE^c^
*Glecaprevir* *+* *Pibrentasvir*	8	X	–	X	–	–
*Glecaprevir* *+* *Pibrentasvir*	12	–	–	–	X	–
*Glecaprevir* *+* *Pibrentasvir*	16	–	X	–	–	X
*Velapatasvir* *+* *Sofosbuvir*	12	X	X	X	X	X^d^

^a^TN, therapienaiv

^b^TE, therapieerfahren im Sinne einer Vorbehandlung mit (pegyliertem) Interferon ± Ribavirin. Zusätzlich für die Therapie mit **Glecaprevir** **+** **Pibrentasvir **auch Vorbehandlung mit Sofosbuvir ± (pegyliertes) Interferon ± Ribavirin, aber ohne zusätzlichen DAA. Zusätzlich für die Therapie mit **Velapatasvir** **+** **Sofosbuvir **auch Vorbehandlung mit Boceprevir o. Telaprevir o. anderen NS3-Proteaseinhibitoren in Kombination mit (pegyliertem) Interferon ± Ribavirin

^c^HCV Gt3 + TE, HCV(Hepatitis-C-Virus)-Genotyp-3-Infektion und therapieerfahren mit (pegyliertem) Interferon ± Ribavirin oder Sofosbuvir + (pegyliertes) Interferon + Ribavirin o. Sofosbuvir + Ribavirin

^d^Gegebenenfalls Hinzunahme von Ribavirin

### Pangenotypisch wirksame DAA-Regime

#### Sofosbuvir/Velpatasvir.

Die Kombination aus dem nukleotidischen Polymeraseinhibitor Sofosbuvir und dem NS5A-Inhibitor Velpatasvir ist pangenotypisch wirksam und wurde im Sommer 2016 in Deutschland zugelassen. Beide DAAs weisen eine hohe antivirale Aktivität sowie eine hohe bis sehr hohe Resistenzbarriere auf. Die empfohlene Dosis beträgt eine Tablette pro Tag (enthält 100 mg Velpatasvir und 400 mg Sofosbuvir) und kann unabhängig von einer Mahlzeit eingenommen werden. Exzellente Viruseradikationsraten von 95–100 % wurden in den Zulassungsstudien für die HCV-Genotypen 1–6 mit diesem Regime beobachtet [13, 14]. In diese Studien waren auch therapieerfahrene Patient*innen mit und ohne kompensierte Leberzirrhose eingeschlossen. Die Therapiedauer beträgt unabhängig vom HCV-Genotyp 12 Wochen, sowohl in therapienaiven als auch in vorbehandelten Patient*innen mit und ohne kompensierte Leberzirrhose. Die Zugabe von Ribavirin kann bei Patient*innen mit HCV-Genotyp-3-Infektion und bereits bestehender Leberzirrhose erwogen werden [15].

#### Glecaprevir/Pibrentasvir.

In diesem koformulierten Regime wird der NS3/4A-Proteaseinhibitor Glecaprevir mit dem NS5A-Inhibitor Pibrentasvir kombiniert. Ebenso wie das oben genannte Regime ist diese Medikamentenkombination aufgrund einer hohen antiviralen Wirksamkeit bei den HCV-Genotypen 1–6 pangenotypisch wirksam und kann daher bei Patient*innen mit und ohne kompensierte Leberzirrhose zur antiviralen Therapie eingesetzt werden. Die empfohlene Dosierung entspricht 3 Tabletten (enthält jeweils 100 mg Glecaprevir und 40 mg Pibrentasvir) einmal am Tag, die Einnahme sollte zusammen mit einer Mahlzeit erfolgen. Die Therapiedauer beträgt bei allen therapienaiven Patient*innen unabhängig vom HCV-Genotyp, mit und ohne kompensierte Leberzirrhose, 8 Wochen. Auch bei Infektionen mit den HCV-Genotypen 1, 2, 4, 5 und 6, die erfolglos mit pegyliertem Interferon ± Ribavirin ± Sofosbuvir vorbehandelt wurden, ist eine 8‑wöchige Therapie ausreichend. Eine Verlängerung der Therapiedauer auf 12 Wochen wird bei in dieser Art vorbehandelten Patient*innen bei bestehender Leberzirrhose empfohlen. Eine Therapieverlängerung auf 16 Wochen sollte bei in dieser Art vorbehandelten HCV-genotyp-3-infizierten Patient*innen unabhängig vom Zirrhosestatus erfolgen [4, 16].

### Genotypspezifisch wirksame Therapieregime

#### Sofosbuvir/Ledipasvir.

In diesem DAA-Regime wird Sofosbuvir mit dem NS5A-Inhibitor Ledipasvir kombiniert. Diese Therapie wurde im November 2014 zur Therapie der chronischen Hepatitis-C-Infektion zugelassen und war damit das erste zugelassene interferonfreie, fix koformulierte Regime. Die empfohlene Dosis beträgt eine Tablette pro Tag, die 90 mg Ledipasvir und 400 mg Sofosbuvir enthält, und kann unabhängig von einer Mahlzeit eingenommen werden. Während Sofosbuvir wie oben beschrieben pangenotypisch mit einer hohen antiviralen Aktivität wirksam ist, weist Ledipasvir *in vitro *eine verminderte antivirale Aktivität bei den HCV-Genotypen 2 und 3 auf und wird daher zur Behandlung dieser Genotypen nicht empfohlen. Zugelassen ist diese Kombination für die antivirale Behandlung von Patient*innen mit den HCV-Genotypen 1, 4 oder 6 (mit und ohne Leberzirrhose) und wird leitliniengemäß lediglich für die Behandlung der HCV-Genotypen 1 und 4 empfohlen [4]. Die Standardtherapiedauer beträgt 12 Wochen. Eine Therapieverkürzung auf 8 Wochen sollte bei therapienaiven, nichtzirrhotischen Patient*innen mit einer HCV-Genotyp-1-Infektion und einer Viruslast (HCV-RNA) von < 6 Mio. IE/mL erfolgen [4]. Bei Patient*innen mit Leberzirrhose und/oder negativen Prädiktoren (z. B. Versagen einer Vortherapie, Thrombozytenzahl < 75.000/nl) kann eine zusätzliche Gabe von Ribavirin erwogen werden [17, 18]. In der klinischen Praxis wird die Kombination Ledipasvir/Sofosbuvir jedoch seit Zulassung der pangenotypisch wirksamen DAA-Regime bei Erwachsenen in Deutschland nur noch selten eingesetzt [4].

#### Grazoprevir/Elbasvir.

Bei diesem Kombinationsregime wird der NS3/4A-Proteaseinhibitor Grazoprevir mit dem NS5A-Inhibitor Elbasvir kombiniert. Beide Substanzen weisen *in vitro *eine hohe antivirale Aktivität gegen die HCV-Genotypen 1 und 4 auf und wurden nach Überprüfung in den Zulassungsstudien zur Behandlung dieser Genotypen zugelassen [19]. Empfohlen wird eine Einmalgabe einer Tablette pro Tag (enthält 100 mg Grazoprevir und 50 mg Elbasvir) entweder mit oder ohne Nahrung. Unabhängig von dem Vorhandensein einer kompensierten Leberzirrhose beträgt die Standardtherapiedauer 12 Wochen. Eine Therapieverkürzung auf 8 Wochen wird aufgrund einer eingeschränkten Datenlage und fehlender Zulassung nicht empfohlen. Allerdings sollte bei der Therapie von HCV-genotyp-1a-infizierten Patient*innen eine Therapieverlängerung auf 16 Wochen in Betracht gezogen werden sowie eine zusätzliche Gabe von Ribavirin zur Senkung des Risikos eines Therapieversagens bei Patient*innen mit einer Ausgangsviruslast von über 800.000 IE/mL. Alternativ kann ein Ausschluss resistenzassoziierter Varianten (RAV) im NS5A-Gen erfolgen. Eine Therapieverlängerung auf 16 Wochen soll auch bei vorhandenem HCV-Genotyp‑4 mit einer Ausgangs-HCV-RNA von > 800.000 IE/ml in Betracht gezogen werden bzw. sollen alternative Therapieregime genutzt werden [19].

## Retherapie bei Vorbehandlung mit interferonfreien DAA-Kombinationen

Bei einem Therapieversagen auf eine Therapie mit einem interferonfreien DAA-Regime unter DAA ist davon auszugehen, dass resistente Virusvarianten, sog. RAVs, selektiert wurden [20]. RAVs können mittels Direktsequenzierung in spezialisierten Laboren bestimmt werden, wobei die Nachweisbarkeit und die Persistenzraten der verschiedenen RAV sich je nach Zeitpunkt der Untersuchung und nach verabreichten DAA zum Teil deutlich unterscheiden. Während der Polymeraseinhibitor Sofosbuvir eine hohe Resistenzbarriere aufweist, sind alle anderen aktuell zugelassenen DAAs durch eine relativ niedrige Resistenzbarriere charakterisiert. Zudem bestehen relevante Kreuzresistenzen zwischen den einzelnen DAAs einer Klasse. Patient*innen, die keine SVR auf eine interferonfreie DAA-Therapie erreicht haben, sollten daher nicht mit dem gleichen Regime erneut behandelt werden. In einer Studie zeigten sich beispielsweise deutlich niedrigere Ansprechraten auf eine verlängerte 24-wöchige Therapie mit Sofosbuvir und Ledipasvir in Patient*innen, die zuvor nicht auf diese Therapie angesprochen haben [4]. Leitliniengemäß sollten Patient*innen mit Versagen einer vorherigen DAA-Therapie der ersten Generation sowie auf eine Therapie mit Sofosbuvir/Velpatasvir unter Berücksichtigung des Vortherapiestatus, der Komedikation (siehe unten) und evtl. Komorbiditäten mit folgender Therapie behandelt werden:

- Voxilaprevir, Velpatasvir und Sofosbuvir für 12 Wochen.

In diesem seit 2017 zugelassenen Regime ist die oben beschriebene Kombination aus Sofosbuvir/Velpatasvir zusätzlich mit einem dritten Wirkstoff, dem NS3/4A-Proteaseinhibitor Voxilaprevir koformuliert. Wie die duale Kombination aus Sofosbuvir und Velpatasvir ist dieses Regime pangenotypisch wirksam und wurde v. a. für schwer therapierbare, mit DAA vorbehandelte Patient*innen entwickelt. In den Phase-3-Zulassungsstudien wurden Patient*innen mit einem Therapieversagen auf interferonfreie DAA-Regime untersucht, hier wurden SVR-Raten von 96–98 % beobachtet. Etwas niedrigere SVR-Raten wurden hier und auch in Beobachtungsstudien v. a. bei HCV-genotyp-3-infizierten Patient*innen, insbesondere mit etablierter Leberzirrhose beobachtet [4, 21–23]. Die empfohlene Dosis beträgt 1 Tablette (enthält 100 mg Velpatasvir und 400 mg Sofosbuvir und 100 mg Voxilaprevir) pro Tag wieder in Verbindung mit einer Mahlzeit. Die Therapiedauer sollte unabhängig vom Genotyp 12 Wochen betragen. Patient*innen mit einem Therapieversagen auf die Gabe von Glecaprevir plus Pibrentasvir wurden nicht in die genannten Zulassungsstudien eingeschlossen, sodass in diesen Fällen formal keine Zulassung für eine Dreifachtherapie mit Velpatasvir/Sofosbuvir und Voxilaprevir besteht. Bei diesen Patient*innen und bei solchen mit einem Nichtansprechen auf eine Dreifachtherapie kann daher vor Therapiebeginn eine Resistenzanalyse und eine Therapieauswahl auf Basis dieser Analyse erwogen werden [4].

## Therapie bei dekompensierter Leberzirrhose

Die dekompensierte Leberzirrhose ist gut definiert und kann klinisch sicher diagnostiziert werden. Aufgrund ihrer pharmakokinetischen Charakteristika und damit einhergehender möglicher Lebertoxizität sind NS3/4A-Proteaseinhibitoren wie Grazoprevir, Glecaprevir und Voxilaprevir bei dekompensierter Leberzirrhose kontraindiziert. Folglich ist man bei dieser Population auf Sofosbuvir und die kombinierbaren NS5A-Inhibitoren wie Velpatasvir und Ledipasvir beschränkt. Die Indikation zur antiviralen Therapie besteht sicher für alle Patient*innen, bei denen mittel- oder langfristig eine Lebertransplantation vermieden werden kann, dies ist typischerweise bei einem MELD-(Model-of-End-stage-Liver-Disease‑)Score von < 20 der Fall [24]. Bei Patient*innen mit bereits bestehender Indikation für eine Lebertransplantation ist die Indikationsstellung jedoch deutlich schwieriger und muss individuell abgewogen werden. Einerseits gibt es Vorteile einer Viruseradikation vor der Transplantation, namentlich ein günstigerer perioperativer Verlauf und eine fehlende Reinfektion des transplantierten Organs. Andererseits gibt es potenzielle Nebenwirkungen der antiviralen Medikamente bei diesen ohnehin schon schwer kranken Patient*innen (insbesondere potenziell erhöhte Infektionsgefahr und vermehrte Laktatazidosen). Hier sollte zunächst eine Absprache mit dem Leber(transplantations)zentrum stattfinden. Optimalerweise sollte im Therapiefall zur Verbesserung der antiviralen Effektivität zusätzlich mit Ribavirin in erniedrigter Initialdosis von 600 mg/Tag behandelt werden.

## Therapie bei hepatozellulärem Karzinom (HCC)

Momentan gibt es aufgrund einer ungenügenden Datenlage keine konkreten Empfehlungen zur HCV-Therapie bei Patient*innen mit hepatozellulärem Karzinom (HCC). Nach Meinung der Autoren ist eine antivirale Therapie sicher indiziert, wenn das HCC kurativ therapiert wird. In der onkologisch palliativen Situation ist es unklar, ob mit einer antiviralen Therapie ein Benefit für die erkrankte Person erreicht werden kann. Hier sollte eine individuelle Entscheidung getroffen werden, die das Tumorstadium, die Lebenserwartung und die Leberfunktion berücksichtigt.

## Therapie bei (prä)terminaler Niereninsuffizienz

Nierenerkrankte Patient*innen mit einer glomerulären Filtrationsrate (GFR) > 30 ml/min/1,73 m^2^ können wie Nierengesunde antiviral behandelt werden [24]. Bei schwerer Nierenfunktionsstörung (GFR < 30 mL min/1,73 m^2^) oder bei Hämodialysepflicht ist Sofosbuvir aufgrund einer ungenügenden Sicherheitsdatenlage nicht zugelassen. Zugelassene antivirale Therapieregime sind hier Grazoprevir/Elbasvir sowie das pangenotypisch einsetzbare Glecaprevir/Pibrentasvir-Regime [18]. Wenn Ribavirin eingesetzt wird, muss hier auf eine adäquate Dosisreduktion geachtet werden. In mehreren Anwendungs- und auch einzelnen prospektiven Studien, die eine Therapie mit Sofosbuvir in Kombination mit Velpatasvir bzw. mit Ledipasvir untersuchten, wurden keine schweren Nebenwirkungen von Sofosbuvir berichtet [4]. Daher, kann leitliniengemäß bei Patient*innen mit einer Kombination zum Beispiel aus dekompensierter Zirrhose und fortgeschrittener Niereninsuffizienz der Einsatz von sofosbuvirbasierten Regimen sinnvoll sein [4].

## Therapie bei Koinfektionen

Im Falle einer Koinfektion mit HIV unterscheiden sich die antiviralen Therapiemöglichkeiten und Ansprechraten nicht wesentlich von der Therapie HCV-monoinfizierter Patient*innen. Es sollten jedoch im Vorfeld ggf. relevante Medikamenteninteraktionen zwischen den DAA und HAART-Medikamenten (hochaktive antiretrovirale Therapie) überprüft werden. Bei der antiviralen Therapie von HBV-koinfizierten Patient*innen sind die Ansprechraten auf eine antivirale HCV-Therapie ebenfalls exzellent, relevante Medikamenteninteraktionen der DAAs mit Entecavir und Tenofovir sind nicht beschrieben. Sehr selten kann es jedoch im Rahmen einer HCV-Therapie zu einer Reaktivierung einer HBV-Infektion kommen, die dann mit einem hochpotenten Nukleotidanalogon suffizient behandelt werden kann [25, 26].

## Therapie von HCV-infizierten Kindern

Für die antivirale Therapie HCV-infizierter Kinder liegen bisher nur eingeschränkte Studienerfahrungen vor, in den letzten Jahren kam es aber auch hier zu Zulassungen verschiedener interferonfreier DAA-Regime für verschiedene Altersklassen [4]. Alle bisherigen Daten deuten auf eine ähnlich gute Verträglichkeit und Wirksamkeit wie bei Erwachsenen hin.

Eine Zulassung besteht für Glecaprevir/Pibrentasvir aktuell für Kinder ab dem 12. Lebensjahr mit gleicher Effektivität, Dosierung und Therapiedauer wie bei Erwachsenen [27]. Auch die Zulassung für 3‑ bis 11-jährige Kinder ist kürzlich erfolgt, hier ist eine Dosisanpassung nach Alter beziehungsweise Gewicht mit einer Granulatformulierung notwendig. Für die ebenfalls pangenotypisch wirksame Kombination aus Sofosbuvir und Velpatasvir besteht eine Zulassung für Kinder ab dem 6. Lebensjahr bei einem Körpergewicht von mindestens 17 kg. Die Dosierung erfolgt körpergewichtsadaptiert. In einer Studie wurden SVR-Raten von 91–100 % erreicht [4]. Für pädiatrische Patient*innen ist zudem die genotypenspezifische wirksame Kombinationstherapie aus Ledipasvir und Sofosbuvir seit 2017 zugelassen. Hier besteht eine Zulassung für Kinder ab 3 Jahren mit alters- und gewichtsadaptierter Dosierung.

## Schwangerschaft und Medikamenteninteraktionen

Aufgrund ungenügender Daten bzgl. des teratogenen Risikos sollte bei Behandlung mit einem DAA und Ribavirin grundsätzlich eine strikte (doppelte) Empfängnisverhütung erfolgen. Eine antivirale Therapie während der Schwangerschaft kann aktuell nicht empfohlen werden.

Sofosbuvir ist insgesamt sehr gut verträglich. Das Nebenwirkungsprofil beinhaltet Übelkeit, Kopfschmerzen und Schlafstörungen. Unter gleichzeitiger Therapie mit dem Antiarrhythmikum Amiodaron wurden lebensgefährliche Bradykardien beobachtet, weshalb die gleichzeitige Einnahme eine Kontraindikation darstellt. Alle zugelassenen NS3/4A-Proteaseinhibitoren (Grazoprevir, Glecaprevir, Voxilaprevir) können zu einem Anstieg der Leberwerte und letztlich zur Notwendigkeit eines Therapieabbruchs führen. Das Nebenwirkungsprofil von Ribavirin ist lange bekannt und gut charakterisiert. Hier sind eine dosisabhängige hämolytische Anämie, Dyspnoe, Reizhusten, Leistungsminderung und Exantheme zu erwarten. Grundsätzlich sollten alle Medikamente, die die Patient*innen in ihrer Komedikation haben, auf mögliche Medikamenteninteraktionen mit antiviralen HCV-Medikamenten geprüft werden. Eine umfassende und leicht bedienbare Datenbank wird zum Beispiel durch das Institut für Pharmakologie der Universität Liverpool bereitgestellt [28].

## Therapieüberwachung und Vorgehen nach der Therapie

Unter Therapie ist keine erneute HCV-RNA-Bestimmung erforderlich, kann aber zur Bestimmung der Compliance durchgeführt werden [4]. Eine Transaminasenbestimmung ist in den meisten Fällen jedoch ausreichend. Eine minimale Restvirämie im Verlauf unter Therapie bzw. am Therapieende ist nicht mit einem Therapieversagen assoziiert und sollte daher nicht zu einem Therapieabbruch oder gar Therapieverlängerung führen. Die Therapieüberwachung sollte sich an den Vorerkrankungen und dem Status der Lebererkrankung orientieren. Wenn Ribavirin eingesetzt wird, sollte der Hämoglobinwert aufgrund der therapieassoziierten hämolytischen Anämie überwacht werden und bezüglich gegebenenfalls notwendiger Dosisreduktion überprüft werden. Eine HCV-RNA-Messung zum Nachweis der SVR sollte 12 Wochen nach Therapieende erfolgen. Aufgrund des verbleibenden HCC-Risikos auch nach erfolgreicher Viruseradikation wird bei Patient*innen mit einer Leberzirrhose, eine (lebenslange) Überwachung (Surveillance) mittels Lebersonographie, gemäß der deutschen Leitlinie zusammen mit der Bestimmung des Alpha-Fetoproteins, alle 6 Monate empfohlen [29].

## Fazit/Ausblick

Die chronische Hepatitis-C-Infektion ist sehr häufig und kann unbehandelt zu schwerwiegenden und potenziell lebensbedrohlichen leberassoziierten Komplikationen führen. Mit den in den letzten Jahren zugelassenen direkt wirkenden antiviralen Medikamenten haben wir nun die Möglichkeit, (fast) alle HCV-Patienten sicher und hocheffizient zu behandeln. Wichtig ist daher eine frühzeitige Erkennung chronischer HCV-Infektionen um potenzielle Komplikationen frühzeitig zu verhindern.
